# Tuning Wetting–Dewetting Thermomechanical Energy for Hydrophobic Nanopores via Preferential Intrusion

**DOI:** 10.1021/acs.jpclett.3c03330

**Published:** 2024-01-19

**Authors:** Luis Bartolomé, Argyrios Anagnostopoulos, Alexander R. Lowe, Piotr Ślęczkowski, Eder Amayuelas, Andrea Le Donne, Michał Wasiak, Mirosław Chora̧żewski, Simone Meloni, Yaroslav Grosu

**Affiliations:** † Centre for Cooperative Research on Alternative Energies (CIC energiGUNE), Basque Research and Technology Alliance (BRTA), Alava Technology Park, Albert Einstein 48, 01510 Vitoria-Gasteiz, Spain; ‡ Institute of Chemistry, University of Silesia, 40-006 Katowice, Poland; § Dipartimento di Scienze Chimiche e Farmaceutiche, Università degli Studi di Ferrara, Via Luigi Borsari 46, I-44121 Ferrara, Italy; ∥ Department of Physical Chemistry, Faculty of Chemistry, University of Łódź, Pomorska 165, 90-236 Łódź, Poland

## Abstract

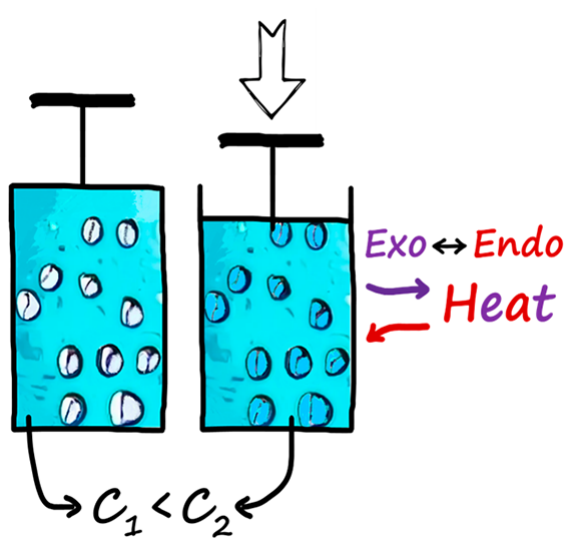

Heat and the work of compression/decompression are among the basic properties of thermodynamic systems. Being relevant to many industrial and natural processes, this thermomechanical energy is challenging to tune due to fundamental boundaries for simple fluids. Here via direct experimental and atomistic observations, we demonstrate, for fluids consisting of nanoporous material and a liquid, one can overcome these limitations and noticeably affect both thermal and mechanical energies of compression/decompression exploiting preferential intrusion of water from aqueous solutions into subnanometer pores. We hypothesize that this effect is due to the enthalpy of dilution manifesting itself as the aqueous solution concentrates upon the preferential intrusion of pure water into pores. We suggest this genuinely subnanoscale phenomenon can be potentially a strategy for controlling the thermomechanical energy of microporous liquids and tuning the wetting/dewetting heat of nanopores relevant to a variety of natural and technological processes spanning from biomedical applications to oil-extraction and renewable energy.

A system consisting of a lyophobic porous material and a nonwetting liquid subjected to sufficient compression/decompression exhibits intrusion/extrusion phenomena^1−5^ relevant for many technological applications such as separation of liquids,^6^ liquid-phase chromatography,^7,8^ porosimetry,^9,10^ energy dissipation,^11,12^ conversion^13,14^ and storage,^15,16^ biological and bioinspired channels,^17^ negative compressibility,^18,19^ and many more.^20,21^ If these heterogeneous lyophobic systems are colloidally stable, it forms a so-called microporous liquid (fluid)^22^—a subclass of liquids with permanent porosity that are promising for gas adsorption technologies.^23^

Despite a broad scope of relevance, the intrusion–extrusion process for nanopores is not fully understood in terms of (i) energy balance,^2,24,25^ (ii) stability,^26^ flexibility,^27^ topology,^28,29^ of a porous material, (iii) nature of intruded/extruded-liquid,^9,16,30,31^ (iv) effect of dissolved gases,^7,32^ and (v) contact electrification.^13,33^ The energies involved in the intrusion–extrusion process are particularly significant in view of the global transition to more sustainable energy conversion solutions,^34,35^ of which the thermal-to-mechanical energy transformation is particularly appealing.^36^

The intrusion–extrusion process is accompanied by thermal effects which are dependent on the properties of the porous material (pore geometry, pore size, etc.^2,3^) and their nonwetting liquid (viscosity, surface entropy, etc.^37,38^) as well as by their interactions.^39^ With this regard, the intrusion–extrusion process of several hydrophobic porous materials, such as silica gels, silicates, zeolites, or metal–organic frameworks (MOFs) has been extensively investigated both experimentally and by molecular dynamic simulations,^2,10,15,24,39−41^ mainly using water as a nonwetting liquid. For example, the pore geometry affects the thermal behavior, i.e., depending on the pore size, the intrusion heat flux can be either exothermic or endothermic whereas the extrusion heat remains exothermic.^2,4^ Using a combination of experimental results and Monte Carlo simulations, this change in the intrusion heat is explained by the number of water molecules exposed to the hydrophobic pore surface.^3^ However, the effect of liquids’ nature on the thermodynamics of the intrusion–extrusion process has not been widely studied, mainly because studies with liquids other than pure water have been focused only on the mechanical behavior, i.e., the intrusion/extrusion pressures.^16,30,31,37,42−45^ The heat of intrusion should not be confused with the heat of spontaneous adsorption of the wetting liquid into the pores as the underlying physics is different.

This paper for the first time reports the thermomechanical properties of microporous fluids by exploring preferential intrusion–extrusion of water from aqueous solutions. Specifically, liquids formed by ZIF-8 MOF and either KBr or *tert*-butanol aqueous solutions were used. We have found that by exploring the enthalpy of solution/dilution, one can tune both the magnitude and the sign of the heat flux generated upon intrusion–extrusion (exothermic or endothermic). Molecular dynamics simulations show that the preferential intrusion of water from the salt solution can be responsible for the observed effect, as it introduces the enthalpy of solution into the intrusion–extrusion cycle. This suggests that preferential intrusion of species from a solution can be exploited to develop a strategy to tune the thermal behavior of the intrusion–extrusion process that is relevant for a broad range of natural and technological systems, including porous fluids.

In Figure 1A, compression of {ZIF-8 + water} fluid is demonstrated up to ∼24 MPa, where above this pressure a sharp decline is observed due to the intrusion of water into the ZIF-8 pores. This sharp change in volume at high pressure results in the storage of ∼10 Joule of mechanical energy per gram of porous material. Upon decompression, water extrudes from the pores at a lower pressure of ∼22 MPa partially releasing the previously stored mechanical energy. Substituting water with an 11.5 wt % of KBr aqueous increases both intrusion and extrusion pressures by ∼20%—Figure 1A. This is expected with respect to previous articles^30,42−45^ that explains this effect via either the increase of the osmotic pressure,^16^ the change of the liquid’s surface tension,^37,46^ the partial^30,45^ or total^42^ suppression of the ions intrusion, or the hydration phenomena during the intrusion–extrusion process.^44^

**Figure 1:**
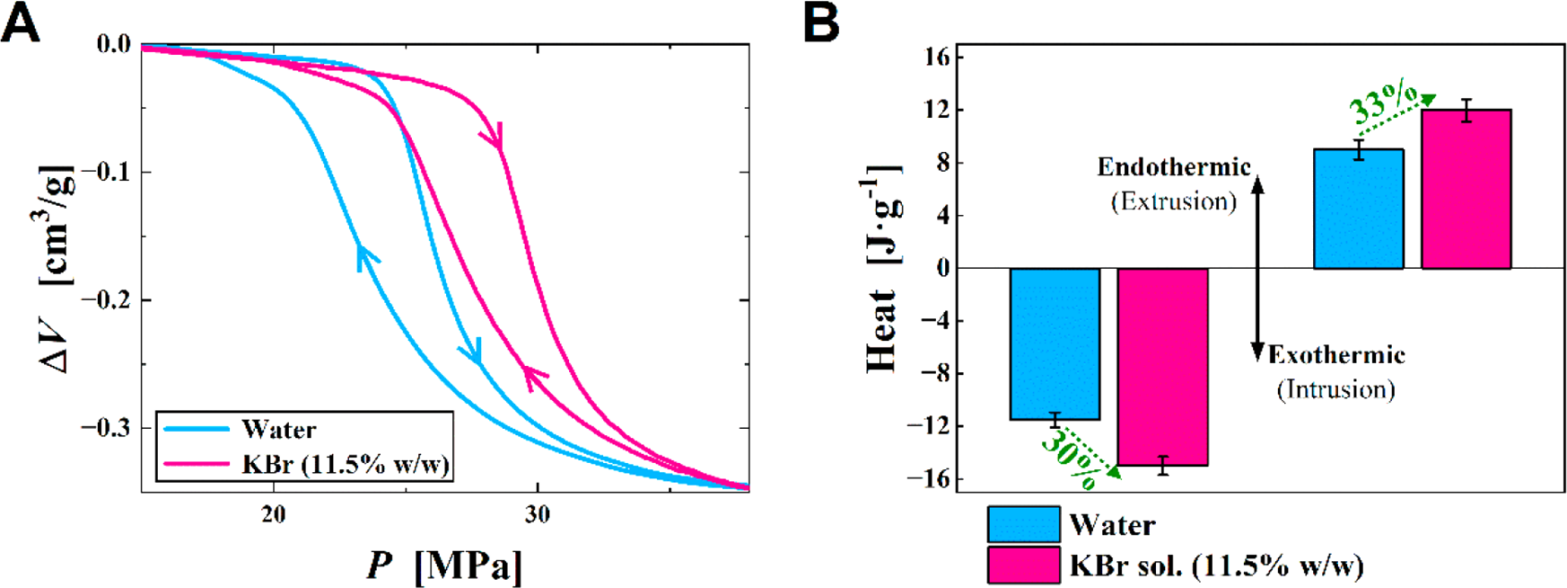
Mechanical and thermal performances for {ZIF-8+water} and {ZIF-8+KBr solution} systems. (A) Compression (intrusion)/decompression (extrusion) *PV*-isotherms. (B) Heats of intrusion and extrusion where they are exothermic in the negative and endothermic in the positive.

For both systems {ZIF-8+water} and {ZIF-8+KBr solution} the intrusion heat is exothermic and the extrusion heat is endothermic. Surprisingly, for the KBr solution case, both heats of intrusion and extrusion are ∼30% higher compared to water.

While some thermodynamic models^3,39,40,47^ were proposed to obtain heat performance during the intrusion–extrusion process, the outcome depends on several factors and their corresponding interactions. Factors such as surface topology and pore size,^2,3^ surface energy,^47^ meniscus formation, temperature,^41^ and chemical interactions, e.g., defect formation,^48^ were explored. To the best of our knowledge, salt solutions were not investigated in terms of the thermal effects of the intrusion–extrusion process. Therefore, a deeper understanding of the recorded rise (Figure 1B) is required.

Molecular dynamics (MD) simulations were conducted to study the intrusion and extrusion of {ZIF-8+water} and {ZIF-8+KBr solution} systems. A so-called *narrow* conical crevice was chosen as a simplistic model of nanoporous material. The introduction of this ideal system allows us to reduce the number of variables that would interfere with the calculation of the heat of intrusion, such as kinetic barriers connected to the activation energy. In fact, this particular geometry has been proven to have a barrierless intrusion process.^49^ With this computational experiment, it is possible to isolate the contribution for the intrusion heat when both water and KBr molecules are allowed to enter inside a nanopore and when the intrusion of KBr is prevented. These simulations allow one to follow the evolution of the intrusion process by quantifying the number of atoms within the crevice (Figure 2A) under varying pressures (Figure 2B), also determining the heat absorbed/released along the process. Three cases were investigated: (i) pure water intrusion; (ii) intrusion of KBr aqueous solution where both water and KBr entered the pore; and (iii) the intrusion of KBr aqueous solution where KBr was prevented from entering the pore, i.e., only water entered the pore.

**Figure 2:**
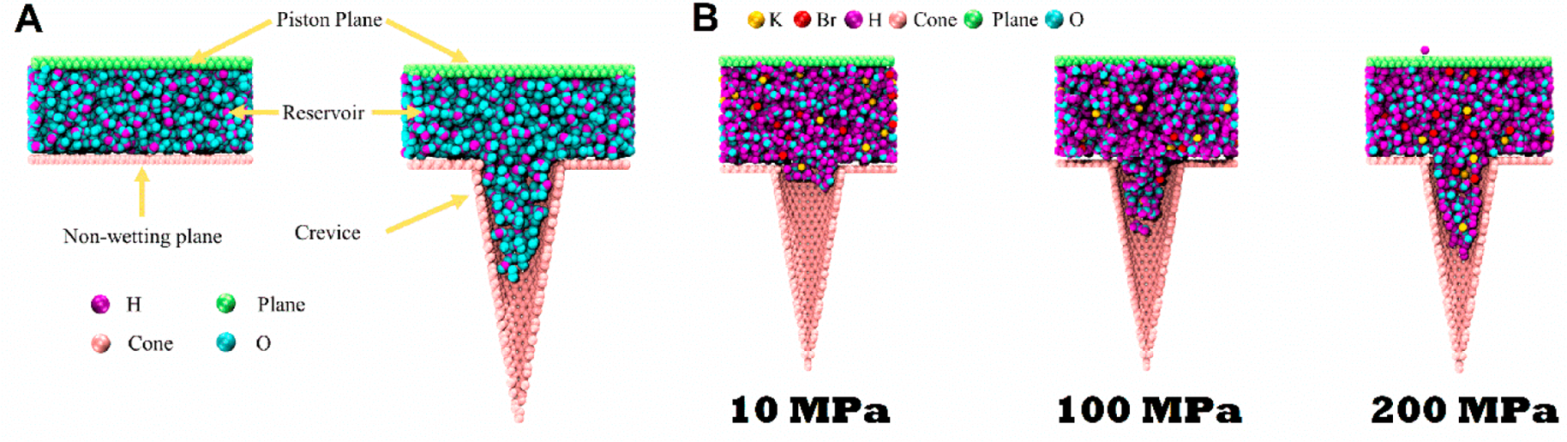
Molecular dynamics of intrusion. (A) Configurations to simulate intrusion where the first system consists of a liquid reservoir situated between two flat planes and the second system where a flat plane is transformed into a plane featuring a 2 nm diameter hole with a crevice (nanocone). (B) Snapshots of the visual molecular dynamics (VMD) for the intrusion of the KBr solution at 10, 100, and 200 MPa.

The MD results show a typical Σ-like profile of the curve of percentage of intruded water molecules vs pressure (Figure 3A), analogous to the experimental liquid volume vs pressure trend. In particular, the amount of water entering the crevice upon pressure increase remained very limited up to 30 MPa, after which intrusion commenced and continued up to 120 MPa, corresponding to the stage where most of the intrusion transpired (Figure 3B). The increased number of molecules in the crevice slowed beyond 120 MPa, showing a plateau up to 240 MPa, the maximum pressure explored in the simulations. This latter branch of the intrusion isotherm corresponds to the liquid compression observed also experimentally (Figure 1A).

**Figure 3:**
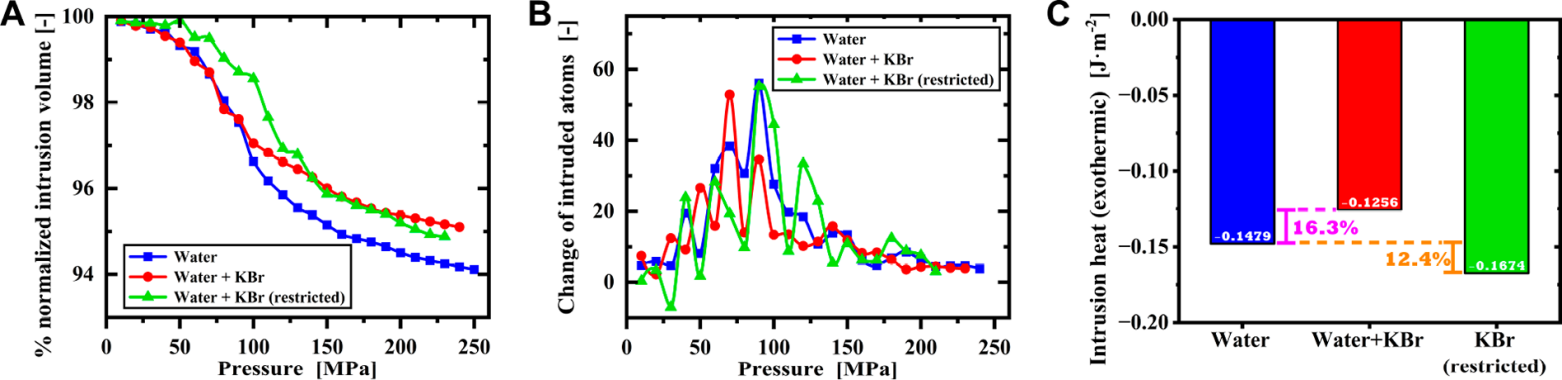
Heat performance of the intrusion process by MD simulations. (A) Percentage of water not intruding into the crevice as a function of pressure. Data are normalized with respect to the system at atmospheric pressure. This is the atomistic counterpart of the experimental variation of volume vs pressure, remarking that computational data cannot be expressed in volume variation per gram of porous material like in experiments because the ratio between the volume of cavities and mass of the solid differs from that of ZIF-8. Data are reported for the three simulated alternatives, i.e., water (blue), KBr solution with salt allowed to enter the crevice (red) and KBr solution with salt prevented from entering the cavity (green). (B) The change in the number of intruded atoms with respect to the previous pressure point depending on the pressure for the three simulated alternatives, i.e., water, free-KBr, and restricted-KBr atoms. (C) The intrusion heat for the three simulated alternatives, i.e., water, free-KBr, and restricted-KBr atoms.

Consistently with experiments, simulations where KBr was prevented from intruding into the pore showed a notable increase in the intrusion pressure of ∼20 MPa (Figure 3A), the same order of magnitude as the experimental value (Figure 1A). Conversely, when KBr was allowed to enter in the crevice, no increment of intrusion pressure was found. This observation suggests that, at least partially, the increase in the intrusion pressure observed experimentally for the {ZIF-8+KBr solution} system is related to the preferential intrusion of water from the KBr solution. Similar shifts of intrusion pressure have been previously reported in the literature.^16^

When KBr is allowed to enter the crevice, the heat of intrusion obtained from MD simulations is 16% less than that of pure water (Figure 3C). This result contradicts the experimental results where the intrusion heat increases significantly using the KBr solution (Figure 1B). Conversely when KBr is prevented from intruding into the crevice, the intrusion heat becomes 12% more exothermic compared with the pure water case (Figure 3C), which is consistent with our experimental observations. These observations are in favor of the hypothesis that the heat rise observed in the experiment is due to the preferential intrusion of water from the KBr aqueous solution into ZIF-8, i.e., the exothermic heat gain during intrusion would be due to getting a more concentrated solution outside the pores since pure water is inside of the pores. The qualitative agreement between experimental and computational heat effects of the salt solution is remarkable when considering the simplistic model of the porous system adopted in this work, which suggests that this effect is more general; i.e., it does not depend critically on the chemistry and topology of the porous system.

Following the hypothesis of preferential water intrusion into the porous media, we propose that the increased exothermic intrusion for an aqueous solution is due to the heat accompanying the variation of concentration of the intruding liquid. Calculation of the enthalpies of dilution qualitatively supports these experimental and numerical observations and suggests that concentrating the KBr solution remaining outside the pores results in additional exothermic effects. While for the extrusion, the KBr solution is diluted by the water leaving the pores, and this solution dilution results in additional endothermic effects. The thermodynamic calculations, from heat of solution data (Supplementary Section 1), show a satisfactory qualitative agreement, but not a quantitative one. The observed quantitative differentiation in the calculated extra heat that comes from the reversible process of concentrating and diluting KBr solution upon preferential water intrusion and extrusion may be due to the preferential intrusion of only some of the salt ions. Indeed, the restriction of certain ions to enter ZIF-8 has a different nature as compared to large molecules that cannot enter due to their size. However, the real picture of preferential intrusion of electrolyte solutions into ZIF-8 may be more complex, requiring deep analysis for each particular case. For example, the preferential intrusion of halide anions against metal cations was numerically demonstrated previously via MD simulations for LiCl aqueous solution, where only Cl^–^ ions intruded into ZIF-8.^50^ Moreover, if ions enter into subnanometer nanopores their concentration is likely to be different from the bulk one as was shown for some hydrophobic zeolites.^51,52^ These factors do not allow quantitative estimation of the resulting heat of intrusion/extrusion based on the solution enthalpy diagram, as was done for large molecules, where the assumption that none of them enter ZIF-8 is reasonable due to their size.

Considering the results of the KBr solution, we then sought to investigate whether the exothermic intrusion result can be changed to endothermic intrusion using a solution with different heats of dilution. For this purpose, aqueous solutions of *tert-*butanol at different concentrations were chosen because (i) its solution enthalpy diagram has a desirable profile which allows more effective tuning of the resulting heat effects (see Supplementary Section 1) and (ii) the molecular size of *tert*-butanol prevents its intrusion into ZIF-8 due to geometrical constraints, thus being free of the possible partial solute intrusion discussed above. Our reasoning is based on *tert*-butanol molecule size, so it cannot enter the pores due to fundamentally geometrical reasons; the polar area of *tert*-butanol is 20.2 Å^2^, so around 5 Å of molecular diameter which is longer than the pore aperture of ZIF-8 of 3.4 Å. This has been confirmed experimentally.^53^ Indeed, free energy MD simulations reveal that it is neither energetically unfavorable for *tert*-butanol to enter ZIF-8 cavities nor kinetically feasible, given the sizable free energy the molecule must overcome to pass from the bulk solution to the confined liquid (see Supplementary Section 2).

We considered several *tert*-butanol aqueous solutions of different concentrations and measured the heats of intrusion and extrusion for each of them. Contrary to KBr, *tert*-butanol aqueous solutions have an endothermic effect in intrusion and exothermic effect in extrusion; i.e., the solute has an effect opposing to the heat developed with pure water. This effect is so prominent that, at greater concentrations, 32 and 50 wt %, the heat of intrusion/extrusion has an opposite sign with respect to the one of pure water (Figure 4). Remarkably, thermodynamic predictions based on the enthalpy of solution indeed predict inversion of the sign of heat of intrusion at the highest *tert*-butanol concentrations (Supplementary Section 1). For *tert*-butanol aqueous solutions, results of thermodynamic analysis are in semiquantitative agreement with experimental results (Figure 4). This is fascinating considering that highly confined water within ZIF-8 cavities might have properties different from those in the bulk case. This improved agreement between thermodynamic calculations and experiments for *tert*-butanol compared to the KBr case may be due to fully selective water intrusion, which is expected both based on size constraints as well as on molecular dynamic simulations. Therefore, in line with the thermodynamic calculations, these experiments demonstrate that one can tune the heat of intrusion/extrusion to be exothermic or endothermic depending on the solution concentration, and this heat change could be related to the variation in the concentration of the solutions due to the preferential intrusion of pure water into the pores. In other words, the nanopores of ZIF-8 act as a filter, concentrating the solution components upon preferential water intrusion and diluting it upon water extrusion.

**Figure 4:**
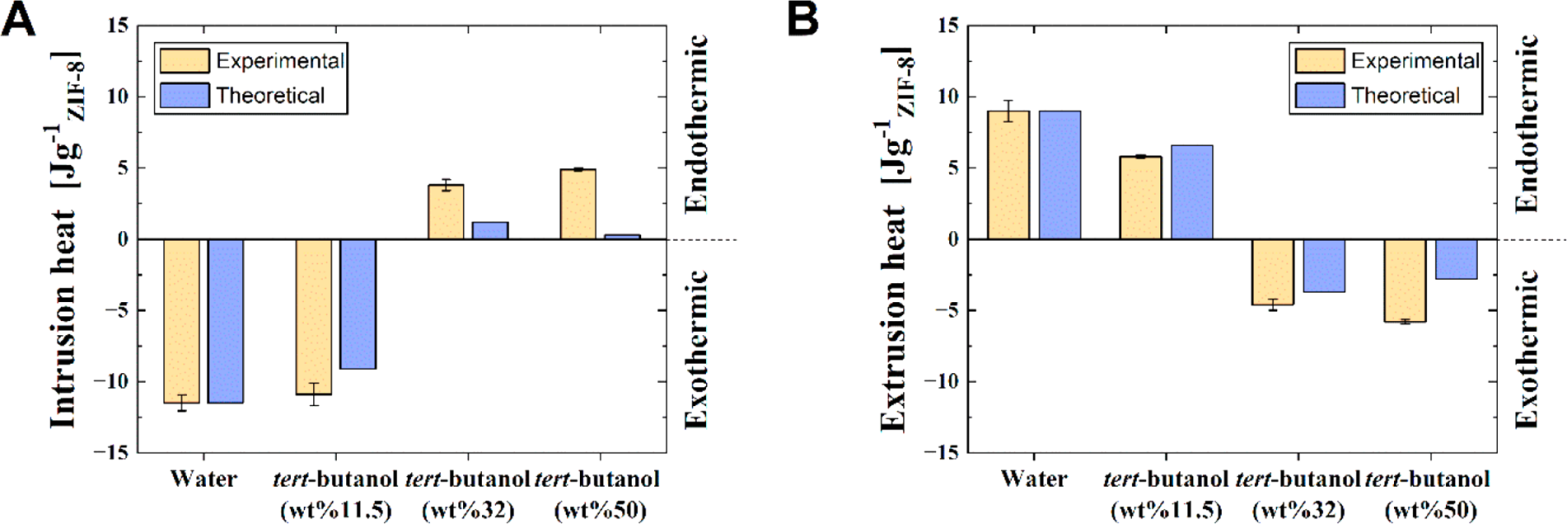
Thermal performance for {ZIF-8 + *tert*-butanol solution}. (A) The heat of intrusion at different solution concentrations both experimental and theoretical. (B) The heat of extrusion at different solution concentrations both experimental and theoretical.

In conclusion, our experimental and theoretical results show that both the mechanical and thermal energies of the intrusion–extrusion process can be tuned via preferential intrusion. After this first proof that the thermomechanical energetics of microporous liquids can be tuned, we demonstrate that by varying the concentration of a *tert*-butanol solution, one can achieve an endothermic or exothermic compression/decompression process. With further development, the obtained results suggest that preferential intrusion of species from a solution into subnanoporous materials can become a strategy for tuning the heat of the intrusion–extrusion process relevant to technological and natural systems.

## Methods

*Materials.* ZIF-8 was purchased from Sigma-Aldrich as Basolite Z1200, CAS #59061-53-9. The smallest pore orifice size of ZIF-8 is 0.34 nm with a surface area of 1800 m^2^ g^–1^. This material had been used in previous investigations.^39,54^ For the 11.5 wt % KBr solution, 100 g of KBr solid was purchased from Chempure and placed in a vacuum oven overnight at 200 °C and 11 mbar. Once the solid was cooled, 11.5 g was weighed into a bottle with 88.5 g of distilled and degassed water. The solution was then stirred and left to equilibrate for 1 h before use. For *tert*-butanol solutions, this compound was purchased from Acros Organics, CAS #75-65-0, and then weighed (11.5, 32, and 50 g) with the corresponding mass of distilled and degassed water (88.5, 68, and 50 g) to obtain the solutions with three different concentrations (11.5, 32, and 50 wt %). The solutions were stirred and left to equilibrate for 1 h before using them.

*Scanning Transitiometer.* A BRG-tech scanning transitiometer, located at the University of Silesia in Katowice, was used to simultaneously record the pressure–volume isotherms and thermograms. A pressure variation of 0.5 MPa min^–1^ was applied, within the pressure range of 5 to 55 MPa, at the isothermal temperature of 298.15 K. A scheme of the equipment (Figure S4) with additional information is in Supplementary Section 3. In these presented experiments, two calorimeter cells, one sample and one reference, each rated for a maximum pressure of 200 MPa, were attached to a single manifold that is connected to a single high-pressure line leading to a high-pressure pump and stepper motor rated for a maximum pressure of 700 MPa. One calorimeter cell was filled with either KBr or *tert*-butanol solution (10 mL of solution) and then sealed at all points with care taken to remove air bubbles from the cells. During and after the intrusion–extrusion cycling, ZIF-8 was verified to be stable—Figure S5 and Figure S6 (see Supplementary Section 4).

*Molecular Dynamics (MD) Simulations.* Using LAMMPS software, we conducted MD simulations, which involved two distinct systems designed with VMD TopoTools. The first system comprised a computational cell containing a liquid reservoir situated between two planes. In contrast, the second system comprised a plane featuring a 2 nm hole with a crevice (nanocone) attached on one side and a liquid reservoir with a plane on the other side (Figure 2A). This approach has the advantage of allowing barrierless intrusion of water into a nonwetting geometry in a narrow cavity space, thereby making it easier to overcome complications arising during such simulations. Both systems had all four lateral planes of the computational cell subjected to periodic boundary conditions. The objective was to calculate the heat of intrusion using the first law of thermodynamics, specifically, the enthalpy. Therefore, in the first system, the total enthalpy corresponded to the heat generated during the compression of the liquid, which was then subtracted from the heat of the crevice system, which encompasses compression and intrusion.

To investigate the impact of KBr on the heat of intrusion, MD simulations of three different configurations were conducted. The first configuration was with water intrusion. The second configuration was with 11.5 wt % KBr solution, where both water and KBr intruded into the crevice. The final third configuration was with 11.5 wt % KBr solution; however, KBr atoms were restricted to intruding in the crevice using the plane command available in LAMMPS. This approach allows us to compare free and preferential (restricted) intrusions of the KBr solution regarding the heat performance to establish if dilution during the intrusion–extrusion process affects the heat of intrusion. A detailed description of these MD simulations is found in Supplementary Section 5.

Finally, to obtain the heat of intrusion of all the simulated alternatives, i.e., the water, the free-KBr and the restricted-KBr, one should bear in mind that the variation in the type of intrusion atoms manifests as a discernible difference in the depth of intrusion. Consequently, the total surface area of the liquid-wetted cone differs for identical pressures in each scenario. To accurately evaluate the heat of intrusion, the calculations were carried out at equivalent depths and plotted for comparable total wetted surface areas.

*Restrained Molecular Dynamics (RMD) Simulations.* The possible intrusion process of a single molecule of *tert*-butanol is achieved by Restrained Molecular Dynamics (RMD) simulations; in this work, we implemented them in a computational sample that consists of a ZIF-8 slab with a 2 × 2 unit cell extension along the *xy* plane (35 × 35 Å) and 7-unit cell extension along the *z*-direction (90 Å) and 4800 water molecules, for a total (ZIF-8 + water) of ∼20000 atoms. The ZIF-8 slab is immersed between two bodies of bulk water, and above and below the water bulk two pistons are used to apply hydrostatic pressure along the *z*-axis.^55^ The simulation runs were carried out applying the NPT *ensemble*, where the fluctuations of the box along the *x*- and *y*-directions were controlled by Tobias–Klein–Martyna barostat;^56^ the applied pressure was set at 25 MPa. The interacting potential, i.e., the force field, of ZIF-8 was the one proposed by Zheng et al.^57^ in combination with the TIP4P/2005 model of water.^58^ This setup was already used and validated in previous works.^18,59^

To calculate the free energy of intrusion, as the starting point of the configuration, a cage, where later the *tert*-butanol will be pushed inside, was filled with water molecules. The *tert*-butanol molecule was placed near the external surface of ZIF-8 and, starting from that position, a linear path, perpendicular to a hexagonal window of the ZIF-8 surface, was pinpointed and followed in order to bring the *tert*-butanol inside the ZIF-8 slab from the bulk solution. The collective variable chosen was the center of mass of the *tert*-butanol molecule to sample the intrusion linear path, applying an RMD coupling constant equal to 15 kcal/mol (see Supplementary Section 6). In combination with RMD, a Parallel Tempering (PT) method was also implemented to enhance the sampling of slow motions which can prevent good sampling of the intrusion process.^60,61^ The implementation of the PT technique allows for faster sampling of slow events, in this case, the rotation of the imidazolate group around the Zn–Im–Zn axis. We choose a range of temperature from 300 to 350 K with a step of 5 K; for the sake of clarity, in the final plots we report only the lowest and the highest temperature, i.e., 300 and 350 K, respectively (see Supplementary Section 2).

## Supporting Information Available

Section 1, calculations of the estimated intrusion and dilution heats; Section 2, free energy profiles for *tert-*butanol intrusion; Section 3, transitiometer description and procedure; Section 4, ZIF-8 stability on intrusion–extrusion tests; Section 5, molecular dynamics simulations; Section 6, restrained molecular dynamics; and supporting references (PDF)
Transparent Peer Review report available (PDF)
