# Supplementary material for: Tuning Wetting–Dewetting Thermomechanical Energy for Hydrophobic Nanopores via Preferential Intrusion
Source: J Phys Chem Lett. 2024 Jan 19;15(4):880–7. doi: 10.1021/acs.jpclett.3c03330 (PMC10839902; doi:10.1021/acs.jpclett.3c03330)
Supplement: Supplementary file 1 — jz3c03330_si_001.pdf [file jz3c03330_si_001.pdf]

Tuning Wetting-Dewetting Thermomechanical Energy for Hydrophobic Nanopores via Preferential Intrusion

Luis Bartolomé,[1,#] Argyrios Anagnostopoulos,[2,#] Alexander R. Lowe,[2,] Piotr Ślęczkowski,[2] Eder Amayuelas,[1] Andrea Le Donne,[3] Michał Wasiak,[4] Mirosław Chorażewski,[2,*] Simone Meloni,[3,*] Yaroslav Grosu[1,*]*

[1] Centre for Cooperative Research on Alternative Energies (CIC energiGUNE), Basque Research and Technology Alliance (BRTA), Alava Technology Park, Albert Einstein 48, 01510 Vitoria-Gasteiz, Spain.

[2] Institute of Chemistry, University of Silesia, 40-006 Katowice, Poland.

[3] Dipartimento di Scienze Chimiche e Farmaceutiche Università degli Studi di Ferrara, Via Luigi Borsari 46, I-44121 Ferrara, Italy.

[4] Department of Physical Chemistry, Faculty of Chemistry, University of Łódź, Pomorska 165, 90-236 Łódź, Poland.

AUTHOR INFORMATION

# These authors contributed equally

* To whom correspondence should be addressed; E-mail: alexander.lowe@us.edu.pl,

miroslaw.chorazewski@us.edu.pl, simone.meloni@unife.it, ygrosu@cicenergigune.com.

Table of contents:

Supplementary Section 1: Calculations of the estimated intrusion and dilution heats

Supplementary Section 2: Free energy profiles for *tert*-butanol intrusion

Supplementary Section 3: Transitiometer description and procedure

Supplementary Section 4: ZIF-8 stability on intrusion-extrusion tests

Supplementary Section 5: Molecular dynamics simulations

Supplementary Section 6: Restrained molecular dynamics

Supplemental References

1. CALCULATIONS OF THE ESTIMATED INTRUSION AND DILUTION HEATS

To calculate the heat of intrusion, what is first needed to be determined is the mass of pure water held within the pores of solid ZIF-8. Then, with a starting ZIF-8 mass of 0.2689 ± 0.0005 g and its specific intrusion volume of 0.34 ± 0.02 $cm^3 \cdot g^{-1}$, the mass of pure water within the porous ZIF-8 from the KBr solution is 0.091 ± 0.005 g. As the calorimetric measuring cell holds 10.0 ± 0.1 g of 11.50 ± 0.01 % w/w KBr solution, then the concentration of the KBr solution increases to 11.60 ± 0.01 % w/w during our scanning transitiometer tests.

Second, the endothermic integral heat of the solution (solution enthalpy) is calculated from a polynomial fit to concentration-dependent experimental data from the literature[1], see Fig. S1. The concentration dependence shows a sharp decrease in the endothermic heat with an increase in solution concentration. Using our two concentration points calculated previously, the molar heat ($\Delta_{sol}H_m$) of KBr solution is 19490 ± 10 $J \cdot mol^{-1}$ for our initial concentration (x_i, 11.5 %) point and 19480 ± 10 $J \cdot mol^{-1}$for the final concentration (x_f, 11.6 %) point. The errors for these molar heats represent root means square results of the fitting curve to the experimental data. As the dilution heat can be calculated from the difference between two values of the integral enthalpy of solution,

$$\Delta_{dil}H_m(x_i \rightarrow x_f) = \Delta_{sol}H_m(x_f) - \Delta_{sol}H_m(x_i) \quad [1]$$

where x_i and x_f represent the initial and final concentrations respectively of the solution with a constant amount of solute[2], then the molar heat of dilution is -10 ± 10 $J \cdot mol^{-1}$ (exothermic heat).

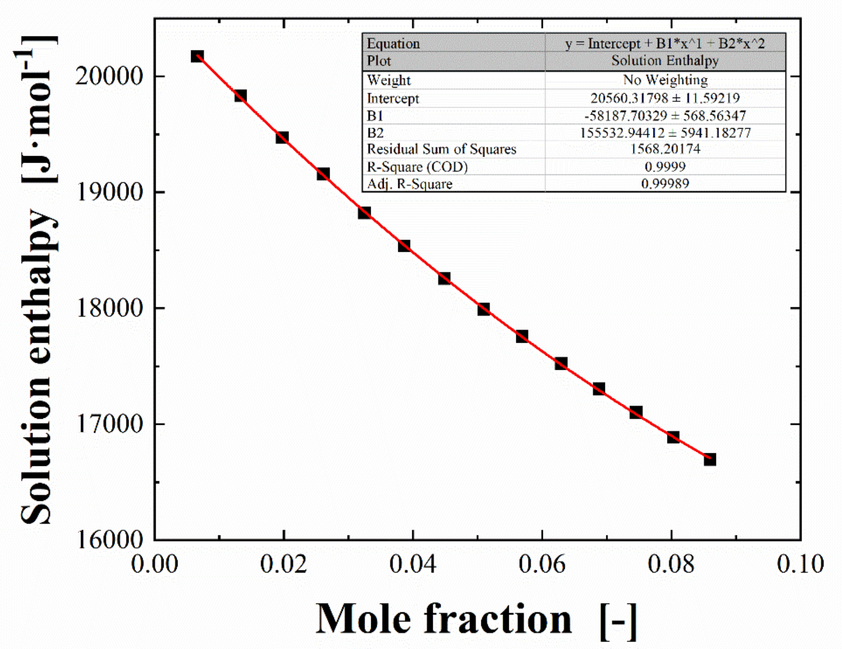

Fig. S1. Solution enthalpy for the KBr solution. The fitting for the heat of the KBr solution depending on the increasing mol fraction of the solution at the temperature of 298 K.

As we have 0.0097 ± 0.0008 mol of KBr in the transitiometer cell, then the absolute heat of dilution will be -0.1 ± 0.1 J. Thus, as 0.2689 ± 0.0005 g of ZIF-8 is used in our scanning transitiometer tests, the obtained dilution heat per mass of ZIF-8 would be -0.4 ± 0.4 $J \cdot g^{-1}{}_{ZIF\text{-}8}$. Considering the heat of intrusion of pure water is -11.5±0.6 $J \cdot g^{-1}{}_{ZIF\text{-}8}$, the additional -0.4± 0.4 $J \cdot g^{-1}{}_{ZIF\text{-}8}$, does not help us reach our experimental result for the intrusion heat of -15.1± 0.7 $J \cdot g^{-1}{}_{ZIF\text{-}8}$ when KBr solution is used for intrusion.

The same process and calculations were conducted for the *tert*-butanol solutions using the corresponding heat of solution fits (see Fig. S2) to experimental data from the literature[3]. The results at different concentrations are summarized in Table S1 and Table S2.

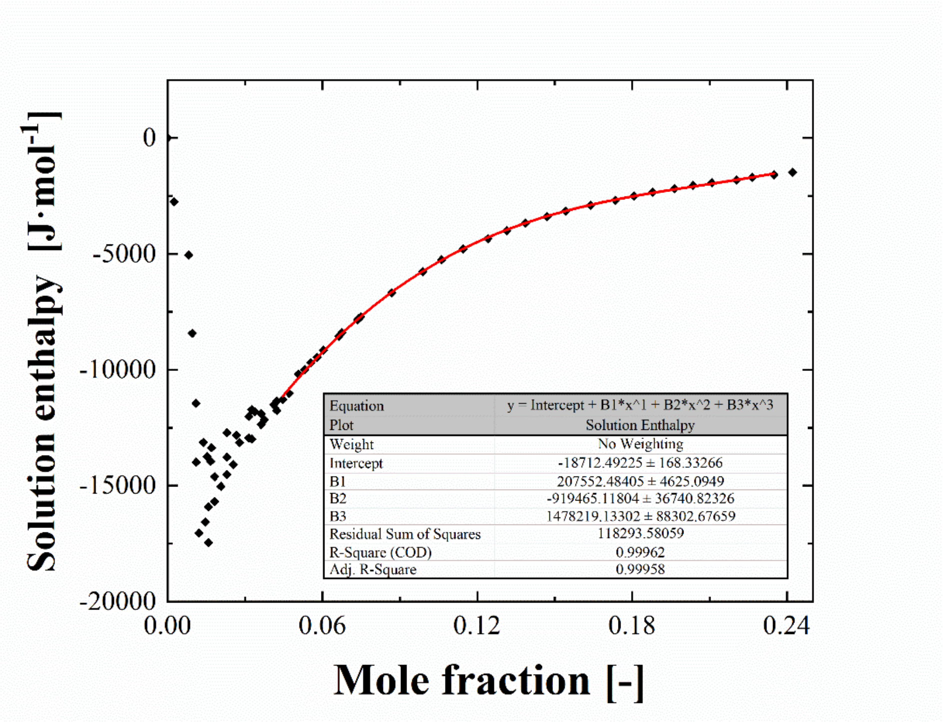

Fig. S2. Solution enthalpy for the *tert*-butanol solution. The fitting for the heat of the *tert*-butanol solution depending on the increasing mol fraction of the solution at the temperature of 298 K.

This quantitative difference between calculations and experiments of the extra heat coming from the reversible process of concentrating and diluting KBr solution upon preferential water intrusion and extrusion may be related to the preferential intrusion of only some of the ions of our salt. This partial preferential intrusion behavior was demonstrated previously *via* MD simulations in the case LiCl aqueous solution, where only Cl^- ions intruded ZIF-8[4]. Therefore, considering the extreme complexity of the intrusion process (dissipative forces and non-equilibrium phenomena), the qualitative agreement of this thermodynamic calculation with the experimental and numerical data is considered satisfactory.

	wt%	Mol fraction	Molal [mol kg^{-1}]	Q_{int}^{Exp} [$J \cdot g^{-1}_{ZIF-8}$]	Q (Dilution) [$J \cdot g^{-1}_{ZIF-8}$]	Q_{int}^{Tho} [$J \cdot g^{-1}_{ZIF-8}$]
**Water**		0	0	-11.5±0.6		-11.5±0.6
**Aqueous KBr**	11.5	0.0193	1.0919	-15.1±0.7	-0.4±0.4	-11.9±0.7
**Aqueous *tert*-butanol**	11.5	0.0306	1.7532	-10.9±0.8	3±2	-9±2
**Aqueous *tert*-butanol**	32	0.1026	6.349	3.9±0.4	13±6	2±6
**Aqueous *tert*-butanol**	50	0.1955	13.4916	4.90±0.08	12±13	1±13

Table S1. Heats of intrusion. The experimental and theoretical heats of intrusion for KBr solution and *tert*-butanol solution at different concentrations.

	wt%	Mol fraction	Molal [mol kg^{-1}]	Q_{ext}^{Exp} [$J \cdot g^{-1}_{ZIF-8}$]	Q (Dilution) [$J \cdot g^{-1}_{ZIF-8}$]	Q_{ext}^{Tho} [$J \cdot g^{-1}_{ZIF-8}$]
**Water**		0	0	9.0±0.7		9.0±0.7
**Aqueous KBr**	11.5	0.0193	1.0919	12.2±0.8	0.4±0.4	9.4±0.8
**Aqueous *tert*-butanol**	11.5	0.0306	1.7532	5.8±0.1	-3±2	7±2
**Aqueous *tert*-butanol**	32	0.1026	6.349	-4.6±0.4	-13±6	-4±6
**Aqueous *tert*-butanol**	50	0.1955	13.4916	-5.8±0.2	-12±13	-3±13

Table S2. Heats of extrusion. The experimental and theoretical heats of extrusion for KBr solution and *tert*-butanol solution at different concentrations.

2. FREE ENERGY PROFILES FOR OF *TERT*-BUTANOL INTRUSION

As you can see from the free-energy profiles of intrusion at 300 K and 350 K (Fig. S3A), the two curves show similar behavior with a minimum after a displacement of around 3-4 Å, which corresponds to a direct contact between the external surface of ZIF-8 and the methyl groups of *tert*-butanol. As the *tert*-butanol is pushed deeper inside, the free energy increases quickly reaching a transition state at ~7 Å when the hydroxyl group is in the middle of the entrance of the ZIF-8 cage. After this transition state, the two curves exhibit slightly different behavior although both curves present a second relative minimum. This second minimum is higher in energy than the first one for both temperatures, but the alcohol-intruded state can stabilize further at 350 K.

To evaluate the probability of the *tert*-butanol molecule entering into the ZIF-8 cage, one determines the energy difference between the initial state and the maximum energy expended during the intrusion process, to calculate the energy barrier to overcome. Using this approach for both temperatures, one observes that the thermal energy available for the process is not sufficient to allow intrusion, due to the heights of the barriers for the process (~23 and ~19 times the available thermal energy at 300 and 350 K, respectively).

To enforce this message, we computed the probability of finding the *tert*-butanol molecule (Fig. S3B) along the intrusion path (Fig. S3C) as the normalized exponential of the two free-energy profiles at both temperatures 300 K and 350 K. As one can see, the alcohol molecule can be found only outside the ZIF-8 and with the maximum probability centered around the corresponding minimum energy of the free-energy profiles.

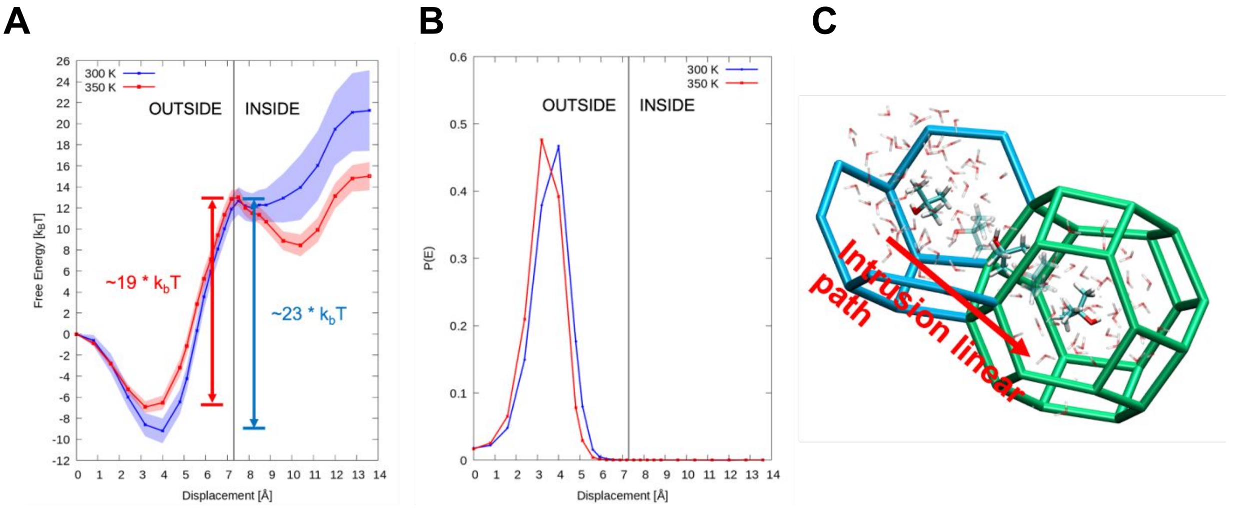

Fig. S3. Infeasibility of tert-butanol molecules from entering the ZIF-8 pores. (A) Free-energy profiles of intrusion at 300 K and 350 K. The vertical grey line is the reference for the transition from outside ZIF-8 to inside the first adjacent cage. The colored shades in the background represent the statistical error in the free energy calculations. **(B)** Probability to find the *tert*-butanol molecule along the intrusion path as the normalized exponential of the two free-energy profiles at 300 K and 350 K. **(C)** Illustrative example of the intrusion path perpendicular to the hexagonal window of ZIF-8. For a displacement equal to 0 Å, the *tert*-butanol is located in the middle of the external surface of ZIF-8 (the light blue structure); when RMD simulations pushed it in the middle of the hexagonal window, the system reaches the maximum value of the energy barrier; finally, the *tert*-butanol is brought to the center of the cage (the green structure). The transparent structures represent the arrangement of water molecules when the *tert*-butanol is outside the ZIF-8 and when they are in a highly confining environment.

3. TRANSITIOMETER DESCRIPTION AND PROCEDURE

The transitiometer is a sensitive twin cell high-pressure heat-flux calorimeter connected to a desktop computer for controlling the state variable of pressure, volume, and temperature using a custom Labview program. The calorimetric block is surrounded by a heating-cooling shield that is thermally isolated from the temperature of the environment. Located inside the calorimetric block are two slots to place calorimetric vessels. One slot is for the sample to be studied (measuring cell) and another for the reference material (reference cell). They are surrounded by 1344 thermocouples (chrom-alumel) and each is mounted differentially in a cylindrical form to detect the differential calorimetric power signal with high sensitivity.

The hydraulic line is composed of a piston pump, pressure detector, manifold, and other high-pressure connecting elements created from 316 SS. The pump is driven by a stepper motor which can be controlled either manually or by computer. This configuration allows the measurement of the volume changes of the system by counting the motor steps. The resolution of the volume detection is ca. $5.79 \pm 0.02 \cdot 10^{-6}$ cm^3 per step. A schematic diagram of the scanning transitiometer used to determine the thermal properties of compressed liquids is shown in Fig. S4.

The scanning transitiometry experiment consists of scanning one of the three variables (Pressure, Volume or Temperature) while the second is held constant. During scanning, the change in the variables and the associated calorimetric signal are simultaneously recorded. For this work, a transitiometer from BGR-Tech was used to record the pressure-volume (PV) isotherms while simultaneously recording the associated thermal effects. From these two changing quantities and the constant variable, two thermodynamic derivatives, thermal and mechanical, are simultaneously recorded for the system under study.

Two configurations have been used for this article. The first one is an isolated configuration where the measuring vessel is the only working vessel and the reference vessel is inactive through isolation from the pressure line and working in a passive reference capacity. The KBr solution tests used this configuration to limit corrosion of the reference cell. The second configuration includes the reference vessel being used in an active capacity, by connecting it to the pressure line, where the pressure in the cell is regulated by the stepper motor and filled with a reference liquid, in this case, water. In both configurations, the measurement cell remains active and both cells can be filled to a maximum of 12 mL of liquid or solution weighed in using a syringe and analytical balance. Both sample and solution must be carefully placed into the cell to limit weighing errors from bubbles and displacement of the liquid.

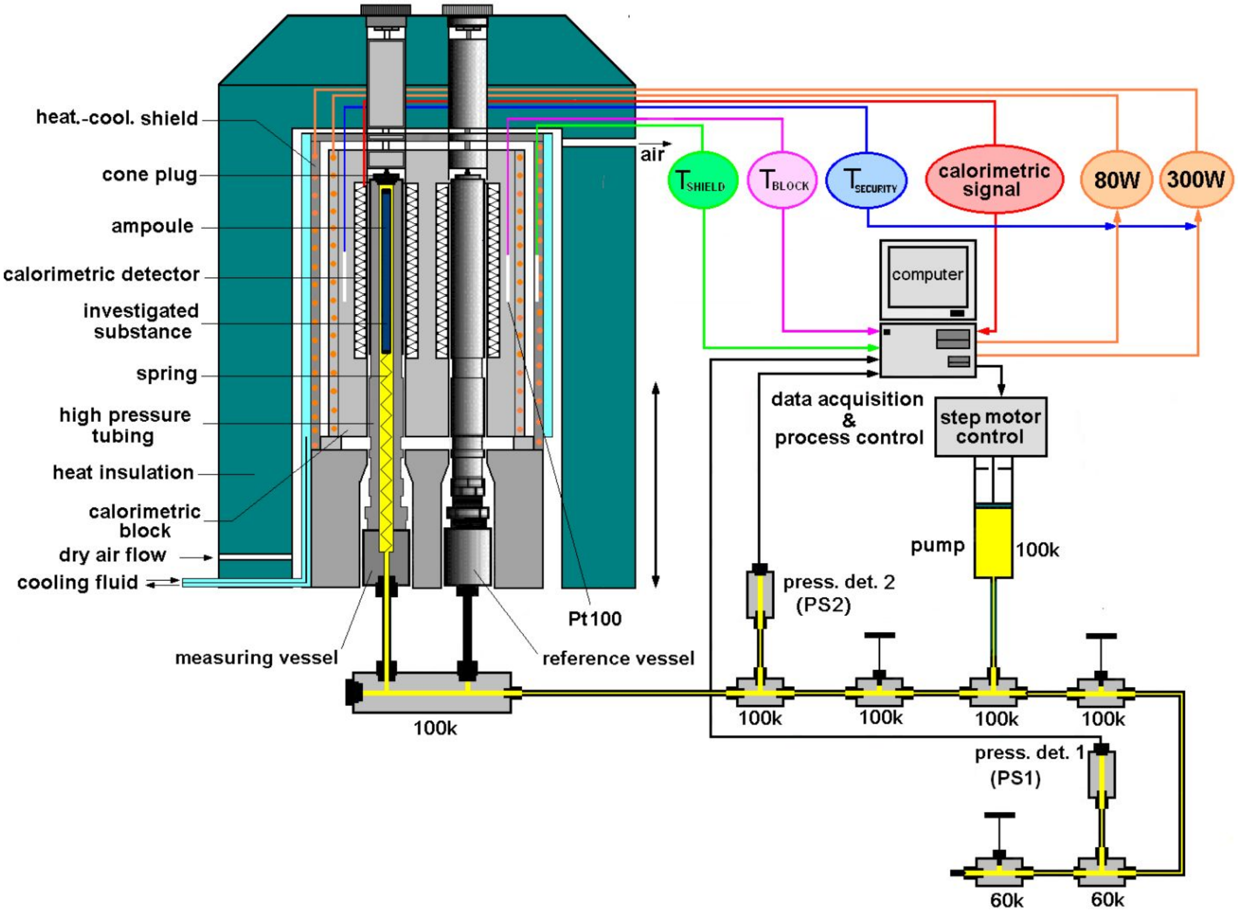

Fig. S4. Scheme of the high-pressure scanning transitiometer.

4. ZIF-8 STABILITY ON INTRUSION-EXTRUSION TESTS

The stability of the porous material, i.e ZIF-8, of our tests using KBr solution as intrusion liquid has been analyzed from two different approaches. Firstly, the steady intrusion-extrusion performance of the ZIF-8 throughout the intrusion-extrusion cycles. Fig. S5A shows the isotherms of three cycles where the intrusion/extrusion pressures and the hysteresis loop shapes remain similar for all the cycles. Moreover, the intrusion volumes of these cycles are comparable to each other and to the intrusion volume when pure water is used as the intrusion liquid (Fig. S5B). Secondly, the chemical stability of the ZIF-8 after testing was investigated. Fig. S6 shows the XRD pattern of both pristine and intrusion/extrusion-tested ZIF-8 after drying. The additional peaks and reflections for the intrusion/extrusion-tested ZIF-8 correspond to the KBr, i.e., after drying both ZIF-8 and KBr are together in the XRD holder but as separated phases, so the peaks and reflections correspond to only KBr.

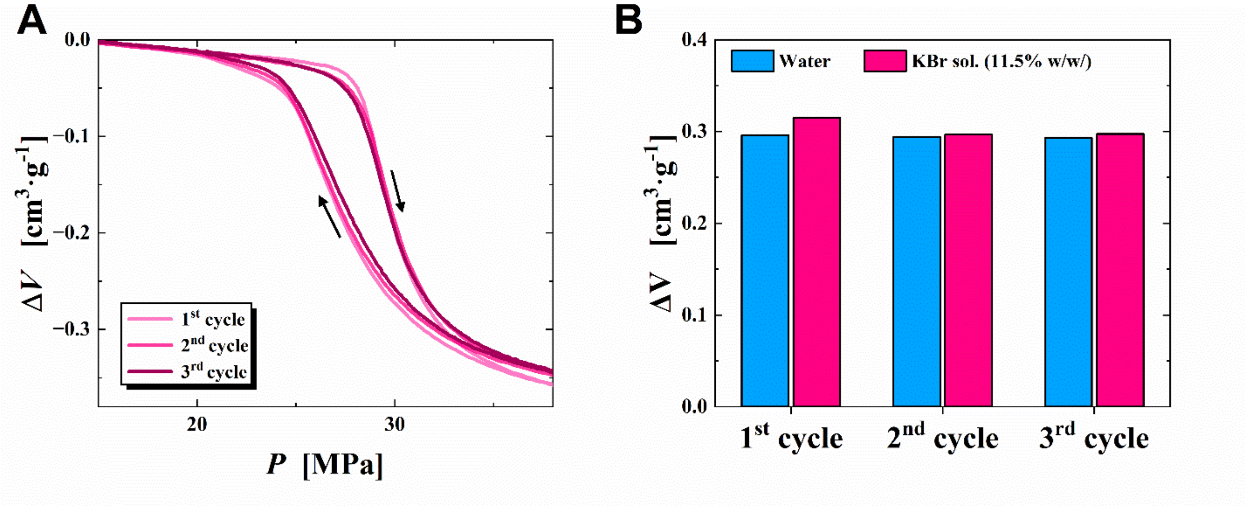

Fig. S5. Intrusion-extrusion performance. (A) PV-isotherms for the cycles of compression/decompression for the system {ZIF-8+KBr solution (11.5% w/w)}. **(B)** Comparison of the intrusion volumes for systems {ZIF-8+water} and {ZIF-8+KBr solution (11.5% w/w)}.

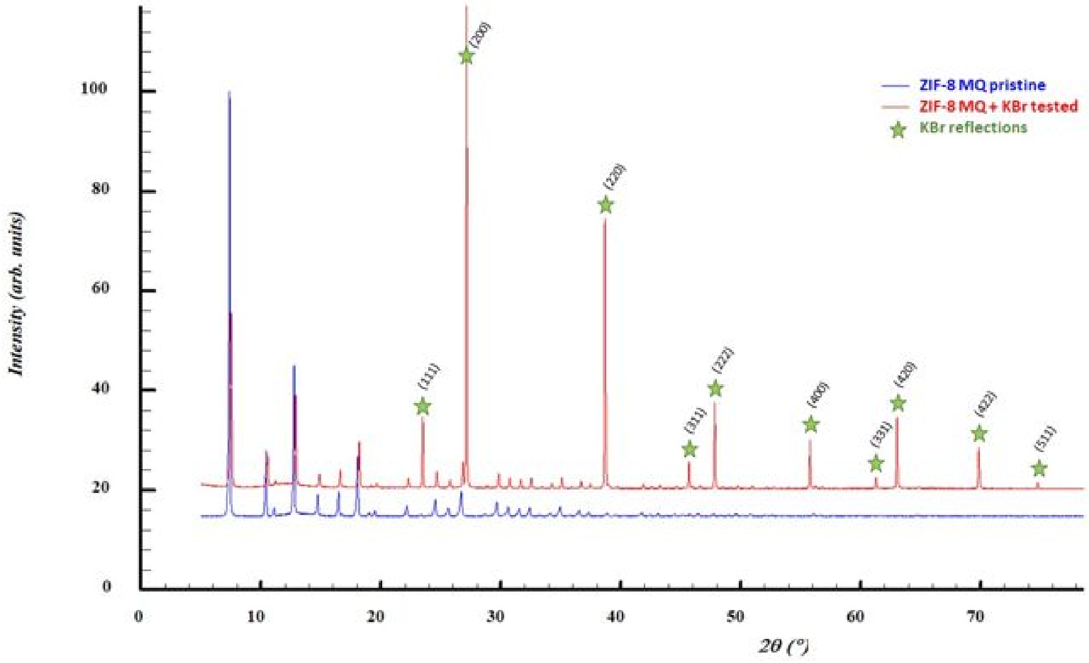

Fig. S6. Comparison of the XRD patterns. The XRD pattern for pristine ZIF-8 (blue) and for ZIF-8 after KBr solution intrusion-extrusion cycling (red). One can see that the structure of ZIF-8 remains intact and the new peaks are related to KBr.

5. MOLECULAR DYNAMICS SIMULATIONS

The following section provides an in-depth example of the computational process applied to water at 298.15 K. Simulations were performed to calculate the system's total energy and volume at 10 MPa intervals. The gathered data were graphically represented to illustrate the relationships between energy, volume, and pressure.

The molecular dynamics (MD) simulations were conducted using LAMMPS software. These MD simulations involved two distinct systems designed with VMD TopoTools. Both systems have all four lateral planes of the computational cell subjected to periodic boundary conditions to calculate the heat of intrusion using enthalpy (first law of thermodynamics). The first system comprises a computational cell containing a water reservoir between two planes and the second system comprises a plane featuring a 2 nm hole with a crevice (nano-cone) jointly with a water reservoir (Fig. 2A). Thus, the enthalpy corresponding to the heat generated during the intrusion can be obtained by subtracting the heat of liquid compression (first system) from the heat of liquid compression when the system has a crevice (second system) which includes compression and intrusion (see Fig. S7). Upon examining the progression of the heat of intrusion against pressure, it is observed that the system, devoid of a cone, exhibits linear behavior, as expected, since the total heat corresponds to compression. Conversely, in pressures below 40 MPa – preceding intrusion onset as identified in Fig. S7– the total heat of the system without a pore closely mirrors that of the pore system, rendering the heat of intrusion effectively null. Beyond 40 MPa, where intrusion incrementally progresses, a corresponding heat increase is noted. The final value, derived at a pressure range with no further observed evolution of intruded atoms, represents the heat of intrusion for water at this particular temperature. A parallel process was executed for KBr.

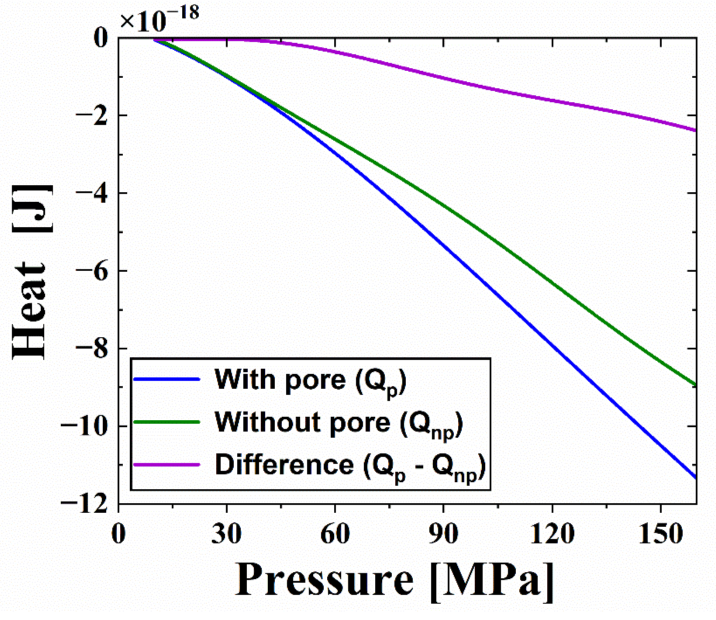

Fig. S7. Heat evolution with pressure. How the heat evolves with the pressure for the system with a pore (blue), without pore (green) and the difference between these two systems (purple).

Initially, the density of water molecules in the reservoir was set to be comparable to that of bulk water, i.e., r_0 = 998.0 kg·m^{-3} at 298.15 K and 1 atm. Both the crevice and the plane were considered rigid objects, and the effect of crevice/surface flexibility on compression and infiltration behavior was assumed to be negligible. The steric and van der Waals (vdW) interactions among the crevice atoms, water molecules, and plane atoms were accounted for using the 12-6 Lennard-Jones (L-J) potential. The water molecules were modeled using the SPC/E potential, which comprises a Coulomb potential between partial point charges on the oxygen and hydrogen atoms and an L-J potential between oxygen atoms. A cutoff distance of 12 Å was selected for the simulations, and the P3M (Particle-Particle Particle-Mesh) technique was employed to handle the long-range Coulomb interactions among water molecules, with a root mean square accuracy of 10^{-4}. The e parameter of the crevice-water interaction, described by the L-J potential, was determined from previous MD simulations of wettability on graphite surfaces and set at a value of 0.015 eV, corresponding to a calculated contact angle of 128º. This contact angle is similar to that of water on hydrophobic MOFs and satisfies the criteria for barrierless intrusion. Since the inner surface of the crevice was non-wetting to water, an external pressure was required for infiltration. Therefore, the system pressure can be adjusted by moving the plane positioned on top of the water reservoir, which essentially acts as a piston. This was achieved through the set-force command in LAMMPS.

Simulations were conducted at pressure intervals of 10 MPa, with each system equilibrated for a total of 6 ns before a 4 ns production run to calculate the total heat using $Q = \Delta E - P\cdot \mathrm{d}V$, where ΔE is the variation of the system total energy, P is the system pressure and dV is the variation of volume (Fig. S8). The computational sequence initiates with the volume determination at a specific pressure. Subsequently, at this given volume, the differential energy (ΔE) relative to the initial value is calculated. Employing these specified pressure and volume metrics, the integral of the pressure in relation to the volume ($P\cdot \mathrm{d}V$) is computed. Upon acquiring both terms, the heat of intrusion (Q) at this specific pressure point is determined using the prescribed formula.

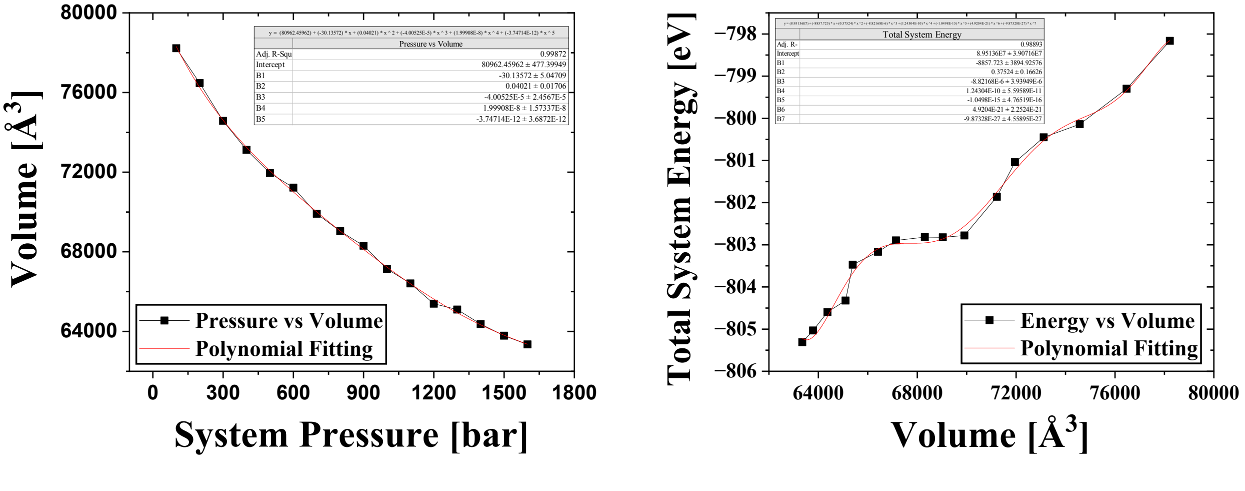

Fig. S8. Polynomial fittings. Relationships between pressure, volume and energy and the corresponding polynomial fittings.

The pressure is assumed to be equal to that applied by the plane and is therefore obtained by summing the force applied on the piston atoms and dividing it by the transversal area. The variation of internal energy is obtained by averaging the total energy of the atoms using the fix ave/time command in LAMMPS, while the variation of volume is nominally defined based on the variation of the position of the piston. The z-coordinate of the fixed water plan/crevice inlet is subtracted from the average coordinate of the piston atoms on the z-axis obtained during data production. To monitor the evolution of the intrusion process, the total average number of atoms inside the cone is calculated during each simulation. The intrusion is considered concluded when the number of intruded atoms does not change significantly for more than three consecutive pressure points.

Finally, to determine the effect of the KBr solution on the intrusion heat, apart from a simulation with water, two additional simulations with 11.5 wt% KBr solution were conducted, one simulation allowing both water and KBr molecules to intrude freely in the crevice and another one simulation restricting the intrusion of the KBr molecules using the plane command available in LAMMPS. This approach enabled us to assess the effect of dilution on the heat of intrusion.

6. RESTRICTED MOLECULAR DYNAMICS SIMULATIONS

In statistical mechanics, a thermodynamic potential is connected to the logarithm of a suitable probability density function in the chosen ensemble. In the present work, the relevant probability density function is expressed as $M(\mathbf{r}^*_{\mathrm{CoM}})$, the probability density that the center of mass of tert-butanol is sampling one point of the linear intrusion path. Introducing the ensemble distribution $m(\boldsymbol{r})$, namely the probability density to find the atoms of the system in position corresponding to the $6N$ dimensional vector $\boldsymbol{r}$, it is possible to express $M(\mathbf{r}^*_{\mathrm{CoM}})$ as:

$$M(\mathbf{r}^*_{\mathrm{CoM}}) = \int d\boldsymbol{r}\, m(\boldsymbol{r})\, \delta(\mathbf{r}_{\mathrm{CoM}} - \mathbf{r}^*) \quad [2]$$

Where $\delta(\ \cdot\ )$ is the Dirac delta function and $\mathbf{r}_{\mathrm{CoM}}$ is the position of the CoM and $\mathbf{r}^*$ is the target value. Under this condition, it is possible to associate the Landau free energy, $G(\mathbf{r}^*_{\mathrm{CoM}})$, with the probability density as following:

$$G(\mathbf{r}^*_{\mathrm{CoM}}) = \ -k_B T \log M(\mathbf{r}^*_{\mathrm{CoM}}) \quad [3]$$

where $k_B T$ is the thermal energy at the experimental temperature T (k_B is the Boltzmann constant). One can compute $M(\mathbf{r}^*_{\mathrm{CoM}})$ by running a long simulation and calculating the histogram of $\mathbf{r}^*_{\mathrm{CoM}}$ along it: the histogram is a good approximation of $M(\mathbf{r}^*_{\mathrm{CoM}})$, from which one can compute the free energy using Eq. 2. However, this approach results as a very inefficient computational procedure, because, with large free energy barriers to intrusion (much higher than the thermal energy $k_B T$), within the typical MD simulation time the system can sample only configurations similar to the initial one.

This problem can be overcome using RMD[5,6]. Inside the RMD framework, a controlled bias is introduced to force the system to explore configurations close to the linear intrusion path. Starting from the Landau free energy Eq. 3 and, for the sake of simplicity, assuming that the ensemble is canonical, we have:

$$m(\mathbf{r}) = \frac{exp\left[{V(\mathbf{r})}/{k_B T}\right]}{\int d\mathbf{r}\, exp\left[{V(\mathbf{r})}/{k_B T}\right]} \quad [4]$$

where $V(\mathbf{r})$ is the force field. Within the canonical ensemble the probability density function of Eq. 2 reads:

$$M(\mathbf{r}^*_{\mathrm{CoM}}) = \frac{\int d\mathbf{r}\, exp\left[{V(\mathbf{r})}/{k_B T}\right] \delta(\mathbf{r}_{\mathrm{CoM}} - \mathbf{r}^*)}{\int d\mathbf{r}\, exp\left[{V(\mathbf{r})}/{k_B T}\right]} \quad [5]$$

The derivative of the free energy is:

$$\frac{dG(\mathbf{r}^*_{\text{CoM}})}{d\mathbf{r}^*_{\text{CoM}}} = -\,k_BT \frac{\int d\mathbf{r}\; exp\left[{V(\mathbf{r})}/{k_BT}\right]{d\left[\delta\left(\mathbf{r}_{\text{CoM}} - \mathbf{r}^*\right)\right]}/{d\mathbf{r}^*_{\text{CoM}}}}{\int d\mathbf{r}\; exp\left[{V(\mathbf{r})}/{k_BT}\right]\delta\left(\mathbf{r}_{\text{CoM}} - \mathbf{r}^*\right)} \qquad [6]$$

$G(\mathbf{r}^*_{\text{CoM}})$ can be computed by numerical integration of ${dG(\mathbf{r}^*_{\text{CoM}})}/{d\mathbf{r}^*_{\text{CoM}}}$. The advantage being that the derivative of free energy can be more easily estimated via atomistic simulations. To achieve this objective, in Eq. 6 one replaces the Dirac delta functions with a smooth Gaussian approximation:

$$\delta\left(\mathbf{r}_{\text{CoM}} - \mathbf{r}^*\right) \sim g_\lambda\left(\mathbf{r}_{\text{CoM}} - \mathbf{r}^*\right) = \sqrt{2\pi k_BT/\lambda}\; exp\left[-\frac{\lambda}{2}{\left(\mathbf{r}_{\text{CoM}} - \mathbf{r}^*\right)^2}/{k_BT}\right] \qquad [7]$$

Here, k_BT/λ is the variance of the Gaussian function, the parameter determining its width, and thus the accuracy of the approximation of the corresponding Dirac delta function. Within the Gaussian approximation the derivative of the free energy, Eq 6, reads:

$$\frac{dG(\mathbf{r}^*_{\text{CoM}})}{d\mathbf{r}^*_{\text{CoM}}} \sim \frac{\int d\mathbf{r}\; \lambda\left(\mathbf{r}_{\text{CoM}} - \mathbf{r}^*\right)\, exp\left\{-\left[V(\mathbf{r}) + \frac{\lambda}{2}\left(\mathbf{r}_{\text{CoM}} - \mathbf{r}^*\right)^2\right]/k_BT\right\}}{\int d\mathbf{r}\; exp\left\{-\left[V(\mathbf{r}) + \frac{\lambda}{2}\left(\mathbf{r}_{\text{CoM}} - \mathbf{r}^*\right)^2\right]/k_BT\right\}} \qquad [8]$$

Thus, the derivative of the free energy can be computed as the expectation value of $\lambda\left(\mathbf{r}_{\text{CoM}} - \mathbf{r}^*\right)$ over the canonical ensemble of a system driven by the so-called augmented potential $V(\mathbf{r}) + \frac{\lambda}{2}\left(\mathbf{r}_{\text{CoM}} - \mathbf{r}^*\right)^2$.

In practice, it is possible to compute ${dG(\mathbf{r}^*_{\text{CoM}})}/{d\mathbf{r}^*_{\text{CoM}}}$ at each point of $\mathbf{r}^*_{\text{CoM}}$ by averaging the observable $\lambda\left(\mathbf{r}_{\text{CoM}} - \mathbf{r}^*\right)$ along MD trajectory driven by the potential $V(\mathbf{r}) + \frac{\lambda}{2}\left(\mathbf{r}_{\text{CoM}} - \mathbf{r}^*\right)^2$. Finally, the ${dG(\mathbf{r}^*_{\text{CoM}})}/{d\mathbf{r}^*_{\text{CoM}}}$ obtained can be numerically integrated by the trapezoid rule.

Error Estimation: Since the free energy is the result of numerical integration of the so-called mean force, which in turn is the expectation of an observable over the biased ensemble average of a suitable observable, the free energy is affected by statistical error. The statistical error on a derived observable $O = O(s)$, with s the directly measured variable. Here the free energy and mean force statistical error, respectively, is usually obtained by error propagation:

$$\delta O^2 = \left(\frac{dO(s)}{ds}\delta s\right)^2 \qquad [9]$$

Here, δs^2 and δO^2 are the variance of s and the estimated of O, respectively.

In free energy calculations thermodynamic integration or analogous techniques, e.g., RMD, where the free energy is obtained by numerical integration of $dG(\mathbf{r}^*_{\mathrm{CoM}}) / d\mathbf{r}^*_{\mathrm{CoM}}$, error propagation leads to a severe overestimation of $dG(\mathbf{r}^*_{\mathrm{CoM}})$:

$$\delta G(\mathbf{r}_j)^2 = \sum_{i=1,j} \frac{G'(\mathbf{r}_i)^2 + G'(\mathbf{r}_{i-1})^2}{2} (\mathbf{r}_i - \mathbf{r}_{i-1})^2 \qquad [10]$$

where $G'(\cdot)$ is a compact notation of $dG(\mathbf{r}^*_{\mathrm{CoM}}) / d\mathbf{r}^*_{\mathrm{CoM}}$.

Here, like in previous works[7,8] we implemented a different approach. We divide the configurations used to estimate $dG(\mathbf{r}^*_{\mathrm{CoM}}) / d\mathbf{r}^*_{\mathrm{CoM}}$ in M smaller sets wherefrom we calculate the corresponding estimates of the mean force $\left\{ \left(dG(\mathbf{r}^*_{\mathrm{CoM}}) / d\mathbf{r}^*_{\mathrm{CoM}} \right)_i \right\}_{i=1,M}$. For each series of free energy gradients, across all values of $\mathbf{r}^*_{\mathrm{CoM}}$, one obtains a set of free energy curves $\left\{ \left(G(\mathbf{r}^*_{\mathrm{CoM}}) \right)_i \right\}_{i=1,M}$, via numerical integration which can be used to directly compute the variance $\delta G(\mathbf{r}_j)^2$ at each value of $\mathbf{r}_j$:

$$\delta G(\mathbf{r}_j)^2 = \frac{1}{M-1} \sum_{i=1,M} \left[G((\mathbf{r}_j))_i - G(\mathbf{r})_J \right]^2 \qquad [11]$$

Here, $G(\mathbf{r}_j)$ is obtained from the numerical integration of $dG(\mathbf{r}^*_{\mathrm{CoM}}) / d\mathbf{r}^*_{\mathrm{CoM}}$ determined by the complete set of simulation data. Correlation effects can be properly considered by using standard techniques such as the block average or the Jack-knife methods[9]. The resulting error values for each point of the two free energy profiles are visualized in Fig. S3A as the two colored shades in the background.

SUPPLEMENTAL REFERENCES

(1) Wüst, J.; Lange, E. Lösungs- Und Verdünnungswärmen von Salzen von Der Äussersten Verdünnung Bis Zur Sättigung. I. *Z. Phys. Chem.* **1925**, *116U* (1), 161–214.

(2) DeVoe, H. Reactions and Other Chemical Processes. In *Thermodynamics and chemistry,* 2nd ed.; University of Maryland, 2020; pp 304-366 (accessed 2023-07-15).

(3) Koga, Y. Excess Partial Molar Enthalpies of Water in Water– *Tert* -Butanol Mixtures. *Can. J. Chem.* **1988**, *66* (12), 3171–3175.

(4) Fraux, G.; Boutin, A.; Fuchs, A. H.; Coudert, F.-X. Structure, Dynamics, and Thermodynamics of Intruded Electrolytes in ZIF-8. *The J. Phys. Chem. C* **2019**, *123* (25), 15589–15598.

(5) Bonella, S.; Meloni, S.; Ciccotti, G. Theory and Methods for Rare Events. *Eur. Phys. J. B* **2012**, *85* (3), 97.

(6) Meloni, S.; Ciccotti, G. Free Energies for Rare Events: Temperature Accelerated MD and MC. *Eur. Phys. J. Spec. Top.* **2015**, *224* (12), 2389–2407.

(7) Janke, W. Statistical Analysis of Simulations: Data Correlations and Error Estimation. *Quantum* **2002**, *10.*

(8) Tortora, M.; Zajdel, P.; Lowe, A. R.; Chorążewski, M.; Leão, J. B.; Jensen, G. V.; Bleuel, M.; Giacomello, A.; Casciola, C. M.; Meloni, S.; Grosu, Y. Giant Negative Compressibility by Liquid Intrusion into Superhydrophobic Flexible Nanoporous Frameworks. *Nano Lett.* **2021**, *21* (7), 2848–2853.

(9) Amabili, M.; Meloni, S.; Giacomello, A.; Casciola, C. M. Activated Wetting of Nanostructured Surfaces: Reaction Coordinates, Finite Size Effects, and Simulation Pitfalls. *J. Phys. Chem. B* **2018**, *122* (1), 200–212.
