# Supplementary material for: Tuning Wetting–Dewetting Thermomechanical Energy for Hydrophobic Nanopores via Preferential Intrusion
Source: J Phys Chem Lett. 2024 Jan 19;15(4):880–7. doi: 10.1021/acs.jpclett.3c03330 (PMC10839902; doi:10.1021/acs.jpclett.3c03330)
Supplement: Supplementary file 2 — jz3c03330_si_002.pdf [file jz3c03330_si_002.pdf]

jz-2023-03330q.R1

Name: Peer Review Information for "Tuning Wetting-Dewetting Thermomechanical Energy for Hydrophobic Nanopores via Preferential Intrusion"

First Round of Reviewer Comments

Reviewer: 1

Comments to the Author

The authors analyze the heat of intrusion/extrusion of pure water, KBr and tert-butanol solutions into ZIF-8 nanopores. The process has been studied both experimentally and through Molecular Dynamics simulations.

I consider the research is well done and that the data provided, both through experiments and Molecular Dynamics simulations, support the main conclusions of the authors.

I have some remarks/questions:

1. Why they authors only measured/simulated one concentration for KBr but three for tert-butanol? Considering that the intrusion/extrusion heat is explained through the change in concentration of the solutions as they enter the nanopores, it is quite curious they only use one concentration for the KBr case.

2. This is related to the previous remark. The authors give a thorough explanation (both in the manuscript and in the SI, of the reason the tert-butanol cannot enter the nanopore (size and energetics restrictions). However this explanation is absent in the case of KBr (desolvation/solvation of K Br ions through entering/exiting the (very narrow) nanopores, perhaps?). Note that one the main results of the work is that the comparison between experiment and simulations indicate that the entrance of K Br ions in the nanopores is resctricted.

3. In figure 3A-C, do the results provided correspond to the average of a number of simulations of the same case? Or just one simulation per case (as it seems it is the case of B)? The bars of figure 3C should follow the colors of figures 3A and 3B. Is figure 3D necessary (other than being the graphical abstract figure)? What information does it provide?

4. Some minor spelling/grammar errors (please be advised I am not a native English speaker):

- Page 8, position 25 of the left margin: "Modelucar"->"Molecular"

- Page 11, position 3 of the left margin: "explored the simulations"->"expored in the simulations"

- Page 13, position 24 of the left margin: "chosen"->"chosen because"?

- Page 13, position 24 of the left margin: "desirable, profile"->"desirable profile"

- Page 14, position 3 of the left margin: "prominent, that at greater"->"prominent that, at greater"

- Page 14, position 34 of the left margin: "expected with both"->"expected both"

- Page 15, position 42 of the left margin: "concentration of solution"->"concentration of a tert-butanol solution"?

Reviewer: 2

Comments to the Author

The manuscript by Bartolome et al. entitled “Tuning wetting-dewetting thermomechanical energy

for hydrophobic nanopores via preferential intrusion” describes experimental and atomistic observations of thermal and mechanical energies of compression/decompression during preferential intrusion of water from aqueous solutions into sub-nanometer pores.

Systems consisting of a lyophobic porous material and a non-wetting liquid exhibiting intrusion/extrusion phenomenon might be relevant for several technological applications.

In this work the authors focus on system based on a MOF framework and KBr/water and tBuOH/water as intrusion/extrusion agents. The experiments have been performed on a model microporous zeolitic imidazolate framewrok 8 (ZIF-8). The authors claim that this effect (preferential water intrusion into the porous media) is due to the enthalpy of dilution of the aqueous solution concentrated upon the preferential intrusion of pure water into the pores.

The experimental observations were supported by molecular dynamics simulations.

Here are some issues which raise some concerns for this referee:

- the authors use various concentrations of KBr in water and previously the authors have shown by MD simulations that for LiCl/water system in ZIF-8 only Cl- ions intruded the pore structure. What would be the scenario for KCl/water system?

- the authors assume that tert-butanol does not penetrate the pores of the MOF, some literature reference or experimental evidence supporting this assumption should be included

This manuscript presents some interesting results on intrusion/extrusion phenomena in porous systems, which could be of interest to physical chemistry community and the readership of J. Phys. Chem. Lett., therefore this referee would recommend publication of this work in this journal after a revision.

Author's Response to Peer Review Comments:

Dr. Yaroslav Grosu
CIC Energigune
Albert Einstein, 48
01510 Miñano, Alava, Spain
email: ygrosu@cicenergigune.com
phone: +34 945 297 108

Dear Editor Prof. Editor,

As requested, we prepared this authors' response letter to the raised comments by the reviewers to our work entitled "**Tuning wetting-dewetting thermomechanical energy for hydrophobic nanopores *via* preferential intrusion**". As you noticed, we have revised the manuscript by modifying the contents based on the comments made by the reviewers. Accordingly, we have uploaded a revised manuscript and SI and additional copies of these files marked with all the changes made during the revision process for review only. The changes are highlighted in yellow.

The point-by-point responses to the reviewers, along with how the manuscript has been revised, can be found below. Moreover, all the required non-scientific changes have been accomplished following your instructions.

We would like to take this opportunity to express our sincere thanks to the reviewers who identified areas of our manuscript that needed corrections or modifications.

Thank you for your time and consideration.

On behalf of all the co-authors,

Alexander Lowe, Mirosław Chorażewski, Simone Meloni, Yaroslav Grosu

Vitoria-Gasteiz, December 22nd 2023

Reviewer 1: The authors analyze the heat of intrusion/extrusion of pure water, KBr and tert-butanol solutions into ZIF-8 nanopores. The process has been studied both experimentally and through Molecular Dynamics simulations.

I consider the research is well done and that the data provided, both through experiments and Molecular Dynamics simulations, support the main conclusions of the authors.

We would like to thank the reviewer for their positive feedback and the interesting corrections and suggestions. You can find below the point-by-point responses.

Point 1. Why they authors only measured/simulated one concentration for KBr but three for tert-butanol? Considering that the intrusion/extrusion heat is explained through the change in concentration of the solutions as they enter the nanopores, it is quite curious they only use one concentration for the KBr case.

> Author's response: We appreciate this remark. The concentration of the KBr solution was chosen to optimize the heat variation following the solution enthalpy diagram (Fig. S1), however, the concentrations of the *tert*-butanol solutions were established to investigate whether the exothermic intrusion result can be changed to endothermic intrusion also following the solution enthalpy diagram (Fig. S2). Thus, the solution enthalpy diagram of KBr suggests that this solution cannot be used to switch the intrusion heat from exothermic to endothermic. We certainly plan to expand the range of solutions and concentrations in the next works.

Point 2. This is related to the previous remark. The authors give a thorough explanation (both in the manuscript and in the SI, of the reason the tert-butanol cannot enter the nanopore (size and energetics restrictions). However this explanation is absent in the case of KBr (desolvation/solvation of K Br ions through entering/exiting the (very narrow) nanopores, perhaps?). Note that one the main results of the work is that the comparison

between experiment and simulations indicate that the entrance of K Br ions in the nanopores is resctricted.

Author's response: Indeed, the restriction of certain ions to enter ZIF-8 has a different nature as compared to large molecules that cannot enter due to their size. To support this hypothesis on the qualitative level, we performed simulations for a model-like system imposing such a restriction. However, the real picture of preferential intrusion of electrolyte solutions into ZIF-8 may be more complex and requires detailed simulations for each particular case. For example, it was demonstrated numerically that in the case of LiCl electrolytes only Cl^- enters ZIF-8, while Li^+ does not.[1] Moreover, if ions enter into sub-nanometer nanopores their concentration is likely to be different from the bulk one as was shown for some hydrophobic zeolites.[2,3] These factors do not allow quantitative estimation of the resulting heat of intrusion/extrusion based on solution enthalpy diagram, as was done for large molecules, where the assumption that none of them enter ZIF-8 is reasonable due to their size. In the original manuscript, we have explained that this extra heat "*comes from the reversible process of concentrating and diluting KBr solution upon preferential water intrusion and extrusion may be due to the preferential intrusion of only some of the salt ions*. Inspired by the comment of the reviewer, we expanded this discussion in the revised manuscript.

New text: The observed quantitative differentiation in the calculated extra heat that comes from the reversible process of concentrating and diluting KBr solution upon preferential water intrusion and extrusion may be due to the preferential intrusion of only some of the salt ions. Indeed, the restriction of certain ions to enter ZIF-8 has a different nature as compared to large molecules that cannot enter due to their size. However, the real picture of preferential

intrusion of electrolyte solutions into ZIF-8 may be more complex requiring deep analysis for each particular case. For example, the preferential intrusion of halide anions against metal cations was numerically demonstrated previously via MD simulations for LiCl aqueous solution, where only Cl- ions intruded ZIF-8.[50] Moreover, if ions enter into sub-nanometer nanopores their concentration is likely to be different from the bulk one as was shown for some hydrophobic zeolites.[51,52] These factors do not allow quantitative estimation of the resulting heat of intrusion/extrusion based on solution enthalpy diagram, as was done for large molecules, where the assumption that none of them enter ZIF-8 is reasonable due to their size.

Point 3. In figure 3A-C, do the results provided correspond to the average of a number of simulations of the same case? Or just one simulation per case (as it seems it is the case of B)? The bars of figure 3C should follow the colors of figures 3A and 3B. Is figure 3D necessary (other than being the graphical abstract figure)? What information does it provide?

Author's response: We appreciate these observations. Regarding the nature of the simulations in Figure 3A-C, each data point presented corresponds to specific pressure points as described in our Supplementary Information. To elaborate, simulations were conducted at pressure intervals of 10 MPa, with each system being equilibrated for a total of 6 ns before a 4 ns production run. This protocol was consistently applied across all cases. To ensure the reliability of our results, we conducted these simulations independently twice for the water systems. This

approach yielded an error margin of approximately 1%. Similarly, for the initial couple of pressure points in the KBr cases, we performed two independent runs, which also resulted in a comparable error margin. Based on these consistent findings, we decided not to extend the number of runs for subsequent pressure points. We acknowledge the discrepancy in the color coding of Figure 3C and thank you for bringing this to our attention. We have corrected this in the revised version of the manuscript to ensure that the colors in Figure 3C align with those in Figures 3A and 3B. Finally, Figure 3d has been removed as well as references in the text.

Point 4. Some minor spelling/grammar errors (please be advised I am not a native English speaker):

- Page 8, position 25 of the left margin: "Modelucar"->"Molecular"

- Page 11, position 3 of the left margin: "explored the simulations"->"expored in the simulations"

- Page 13, position 24 of the left margin: "chosen"->"chosen because"?

- Page 13, position 24 of the left margin: "desirable, profile"->"desirable profile"

- Page 14, position 3 of the left margin: "prominent, that at greater"->"prominent that, at greater"

- Page 14, position 34 of the left margin: "expected with both"->"expected both"

- Page 15, position 42 of the left margin: "concentration of solution"->"concentration of a tert-butanol solution"??

Author's response: We are grateful for all these spelling/grammar suggestions that have been considered in the revised manuscript. They are yellow highlighted in the marked copy for an easy review.

References

(1) Fraux, G.; Boutin, A.; Fuchs, A. H.; Coudert, F.-X. Structure, Dynamics, and Thermodynamics of Intruded Electrolytes in ZIF-8. *J. Phys. Chem. C* **2019**, *123* (25), 15589–15598.

(2) Arletti, R.; Ronchi, L.; Quartieri, S.; Vezzalini, G.; Ryzhikov, A.; Nouali, H.; Daou, T. J.; Patarin, J. Intrusion-Extrusion Experiments of MgCl2 Aqueous Solution in Pure Silica Ferrierite: Evidence of the Nature of Intruded Liquid by in Situ High Pressure Synchrotron X-Ray Powder Diffraction. *Microporous and Mesoporous Mater.* **2016**, *235*, 253–260.

(3) Confalonieri, G.; Ryzhikov, A.; Arletti, R.; Nouali, H.; Quartieri, S.; Daou, T. J.; Patarin, J. Intrusion-Extrusion of Electrolyte Aqueous Solutions in Pure Silica Chabazite by in Situ High Pressure Synchrotron X-Ray Powder Diffraction. *J. Phys. Chem. C* **2018**, *122* (49), 28001-28012.

Vitoria-Gasteiz, December 22nd 2023

Reviewer 2: The manuscript by Bartolome et al. entitled "Tuning wetting-dewetting thermomechanical energy for hydrophobic nanopores via preferential intrusion" describes experimental and atomistic observations of thermal and mechanical energies of compression/decompression during preferential intrusion of water from aqueous solutions into sub-nanometer pores.

Systems consisting of a lyophobic porous material and a non-wetting liquid exhibiting intrusion/extrusion phenomenon might be relevant for several technological applications.

In this work the authors focus on system based on a MOF framework and KBr/water and tBuOH/water as intrusion/extrusion agents. The experiments have been performed on a model microporous zeolitic imidazolate framewrok 8 (ZIF-8). The authors claim that this effect (preferential water intrusion into the porous media) is due to the enthalpy of dilution of the aqueous solution concentrated upon the preferential intrusion of pure water into the pores.

The experimental observations were supported by molecular dynamics simulations.

We would like to thank the reviewer for positively assessing this work and recognizing its relevance for potential technological applications as well as the interesting observations and suggestions. You can find below the point-by-point responses.

Point 1. The authors use various concentrations of KBr in water and previously the authors have shown by MD simulations that for LiCl/water system in ZIF-8 only Cl- ions intruded the pore structure. What would be the scenario for KCl/water system?

Author's response: We agree with this comment since we believe that additional work in the intrusion-extrusion of aqueous solutions needs to be carried out to understand and explain the underlying mechanisms occurring during these intrusion/extrusion processes. Thus, future works are promising where, as pointed out by the reviewer, the KCl solutions would be intriguing to compare with

KBr and LiCl solutions. Indeed, the restriction of certain ions to enter ZIF-8 has a different nature as compared to large molecules that cannot enter due to their size. To support this hypothesis on the qualitative level, we performed simulations for a model-like system imposing such a restriction. However, the real picture of preferential intrusion of electrolyte solutions into ZIF-8 may be more complex and requires detailed simulations for each particular case. For example, it was demonstrated numerically that in the case of LiCl electrolytes only Cl^- enters ZIF-8, while Li^+ does not.[1] Moreover, if ions enter into sub-nanometer nanopores their concentration is likely to be different to the bulk one, as was shown for some hydrophobic zeolites.[2,3] These factors do not allow quantitative estimation of the resulting heat of intrusion/extrusion based on solution enthalpy diagram, as was done for large molecules, where the assumption that none of them enter ZIF-8 is reasonable due to their size. In the original manuscript, we have explained that this extra heat "*comes from the reversible process of concentrating and diluting KBr solution upon preferential water intrusion and extrusion may be due to the preferential intrusion of only some of the salt ions*. Inspired by the comment of the reviewer, we expanded this discussion in the revised manuscript. We are keen to examine other electrolytes in the future including KCl.

New text: The observed quantitative differentiation in the calculated extra heat that comes from the reversible process of concentrating and diluting KBr solution upon preferential water intrusion and extrusion may be due to the preferential intrusion of only some of the salt ions. Indeed, the restriction of certain ions to enter ZIF-8 has a different nature as compared to large molecules that cannot enter due to their size. However, the real picture of preferential intrusion of electrolyte solutions into ZIF-8 may be more complex requiring deep analysis for each particular case. For example, the preferential intrusion of halide anions

against metal cations was numerically demonstrated previously via MD simulations for LiCl aqueous solution, where only Cl- ions intruded ZIF-8.[50] Moreover, if ions enter into sub-nanometer nanopores their concentration is likely to be different from the bulk one as was shown for some hydrophobic zeolites.[51,52] These factors do not allow quantitative estimation of the resulting heat of intrusion/extrusion based on solution enthalpy diagram, as was done for large molecules, where the assumption that none of them enter ZIF-8 is reasonable due to their size.

Point 2. The authors assume that tert-butanol does not penetrate the pores of the MOF, some literature reference or experimental evidence supporting this assumption should be included.

Author's response: We understand this concern. Our reasoning is based on *tert*-butanol molecules size, so it cannot enter the pores due to fundamentally geometrical reasons, the polar area of *tert* butanol is 20.2 Å^2, so around 5 Å of molecular diameter which is longer than the pore aperture of ZIF-8 of 3.4 Å. This has been confirmed experimentally.[4] In this reference, the authors wrote: "*As shown in Figure 5a, all alcohols are detectable except tert-butanol of which the molecular size is too bulky to penetrate into the cage of ZIF-8.*" This will be added to the text to be clearer for the readers.

Text added: Our reasoning is based on *tert*-butanol molecule size, so it cannot enter the pores due to fundamentally geometrical reasons, the polar area of *tert*-butanol is 20.2 Å^2, so around 5 Å of molecular diameter which is longer than the pore

aperture of ZIF-8 of 3.4 Å. This has been confirmed experimentally.[53]

This manuscript presents some interesting results on intrusion/extrusion phenomena in porous systems, which could be of interest to physical chemistry community and the readership of J. Phys. Chem. Lett., therefore this referee would recommend publication of this work in this journal after a revision.

We want to thank the reviewer once again for endorsing this work as attractive to the physical chemistry community and for recommending the publication of this manuscript in The Journal of Physical Chemistry Letters.

References

(1) Fraux, G.; Boutin, A.; Fuchs, A. H.; Coudert, F.-X. Structure, Dynamics, and Thermodynamics of Intruded Electrolytes in ZIF-8. *J. Phys. Chem. C* **2019**, *123* (25), 15589–15598.

(2) Arletti, R.; Ronchi, L.; Quartieri, S.; Vezzalini, G.; Ryzhikov, A.; Nouali, H.; Daou, T. J.; Patarin, J. Intrusion-Extrusion Experiments of MgCl2 Aqueous Solution in Pure Silica Ferrierite: Evidence of the Nature of Intruded Liquid by in Situ High Pressure Synchrotron X-Ray Powder Diffraction. *Microporous and Mesoporous Mater.* **2016**, *235*, 253–260.

(3) Confalonieri, G.; Ryzhikov, A.; Arletti, R.; Nouali, H.; Quartieri, S.; Daou, T. J.; Patarin, J. Intrusion-Extrusion of Electrolyte Aqueous Solutions in Pure Silica Chabazite by in Situ High Pressure Synchrotron X-Ray Powder Diffraction. *J. Phys. Chem. C* **2018**, *122* (49), 28001-28012.

(4) Tu, M.; Wannapaiboon, S.; Khaletskaya, K.; Fischer, R. A. Engineering Zeolitic-Imidazolate Framework (ZIF) Thin Film Devices for Selective Detection of Volatile Organic Compounds. *Adv Funct Mater* **2015**, *25* (28), 4470-4479.
